# Simplified Characterization of Anisotropic Yield Criteria for an Injection-Molded Polymer Material

**DOI:** 10.3390/polym15234520

**Published:** 2023-11-24

**Authors:** Sharlin Shahid, Eskil Andreasson, Viktor Petersson, Widaad Gukhool, Yuchi Kang, Sharon Kao-Walter

**Affiliations:** 1 Department of Mechanical Engineering, Blekinge Institute of Technology, 371 79 Karlskrona, Sweden; widaad.gukhool@gmail.com; 2 Tetra Pak, 223 55 Lund, Sweden; eskil.andreasson@tetrapak.com (E.A.); viktor.petersson@tetrapak.com (V.P.); 3 Faculty of Mechanical and Electrical Engineering, Kunming University of Science and Technology, Kunmming 650500, China; yuchi.kang@kust.edu.cn; 4 Department of Mathematics and Natural Sciences, Blekinge Institute of Technology, 371 79 Karlskrona, Sweden; sharon.kao-walter@bth.se; 5 College of Engineering, Shanghai Polytechnic University, Shanghai 201209, China

**Keywords:** polyethylene, anisotropic yield criteria, finite element model, injection molding

## Abstract

Injection-molded polyethylene plates exhibit highly anisotropic mechanical behavior due to, e.g., the uneven orientation of the polymer chains during the molding process and the differential cooling, especially in the thickness direction. Elastoplastic finite element modeling of these plates in particular is used with isotropic yield criteria like von Mises, trading off accuracy in favor of simpler constitutive characterization and faster solution. This article studies three different anisotropic yield criteria, namely, Hill 1948, Barlat Yld91, and Barlat Yld2004-18P, for the finite element modeling of low-density polyethylene (LDPE) at large uniaxial tensile deformation and compares the accuracy and computation time with von Mises. A simplified calibration technique is investigated to identify the constitutive parameters of the studied Barlat group yield criteria. The calibration process is simplified in the sense that only uniaxial tensile tests with digital image correlation measurements are used for the calibration of all the yield criteria studied in this article, although a standard calibration procedure for the Barlat group yield criteria requires additional material testing using more demanding test setups. It is concluded that both Barlat Yld91 and Barlat Yld2004-18P yield criteria can be calibrated with only a few tensile tests and still capture anisotropy in deformation–stress–strain at different levels of accuracy.

## 1. Introduction

Low-density polyethylene (LDPE) is a polymer of significant importance due to its versatile applications, particularly in the realm of liquid food packaging. Understanding the mechanical properties of this polymer is crucial for optimizing its performance. In the context of thin polymers and polymer plates used in packaging, the isotropic mechanical properties, including elasticity, plasticity, and damage, are commonly characterized using uniaxial tensile testing, encouraged by its simplicity. However, LDPE and similar polymers exhibit highly anisotropic behavior, meaning the material properties vary across different material orientations. This anisotropy arises from factors such as non-uniform material flow and cooling, among others [1]. Specifically, during the filling stage of the injection-molding cycle, the inner core of the polymer experiences relatively slow cooling, while the outer surface in contact with the mold surfaces undergoes rapid heat exchange, resulting in the formation of a thin skin layer on the polymer. In contrast with the skin layer, which exhibits no preferred orientation of the polymer chains and is generally insignificant in terms of mechanical properties, there exists a distinct variation in properties between the skin and bulk layers of the polymer. Adjacent layers of the polymer melt flow at varying velocities, leading to varying shear stresses. These shear stresses, in turn, cause the polymer chains to align in the direction of the polymer flow, resulting in materials with pronounced anisotropy and distinct mechanical properties in different orientations [2]. This alignment gives rise to a highly oriented shear layer, which is commonly referred to as being in a state of frozen strain [2,3,4,5]. Furthermore, these materials are also expected to be inhomogeneous in the thickness direction.

In order to model orthotropic plastic behavior, many elastoplastic orthotropic yield criteria have been proposed. Among these, the Hill 1948 [6], Hill 1990 [7], Barlat Yld89 [8], Barlat Yld91 [9], Barlat Yld2004-18P [10], BBC2005, and BBC2008 [11] criteria are commonly used in numerical methods for analyzing sheet-metal-forming processes. Extensive studies comparing different yield criteria for the sheet metal formation of materials such as steel, aluminum, and titanium can be found in the literature [12,13]. Polymers like LDPE differ from metals in molecular structure and can withstand very high strain before strain localization and failure [1,14]. Limited attempts have been made to apply these models in polymer simulation, and there is currently no comprehensive comparison of their accuracy available in the literature for injection-molded polymers. Bazzi et al. investigated thermoformed components of acrylonitrile butadiene styrene (ABS) using the Barlat Yld89 model to predict thickness distribution [15]. The potential of using local anisotropy for toughening semi-crystalline polymeric materials was also explored. In another study, the semi-crystalline polymeric matrix material of a composite was modeled using anisotropic Hill plasticity with rate-dependent hardening [16]. Erp et al. simulated high-density polyethylene (HDPE) and polypropylene (PP) using the Hill 1948 anisotropic model [17]. These studies reported improved simulation accuracy when adopting anisotropic yield criteria compared with isotropic ones. Most recently, some authors of this article examined the accuracy of the Hill 1948 and Barlat Yld2004-18P models in simulating an LDPE tensile test. They compared the force response, stress and strain anisotropy response, and full-field strain distribution between physical tensile tests and their finite element simulation in Abaqus, demonstrating improved simulation predictions [18].

However, in the context of finite element simulation, LDPE is often assumed to be isotropic in its material behavior [14,19,20], although this assumption yields inaccurate finite element model prediction. Widely used isotropic yield functions are Tresca and von Mises. A major reason for this is the cost and time associated with anisotropic yield criteria characterization and subsequently solving the finite element models (FE models). For example, to calibrate Barlat Yld2000–2D anisotropic parameters for an FE model input, in addition to uniaxial tensile tests in a 0°, 45°, and 90° material orientation, a bulge test has to be performed to identify the bi-axial material response [21]. Bulge testing needs special test-rig and 3D digital image correlation (DIC) systems, which are expensive and absent in most material testing labs. For polymers, different cruciform-designed specimens are used for biaxial tensile testing, which also requires a specialized test rig [22]. It is relevant to mention that in addition to the above-mentioned elastoplastic constitutive modeling approach for polymers, there are other anisotropic viscoelastic–viscoplastic models that have been successfully implemented in many studies [23,24,25].

In the present paper, the aim was to explore and analyze the applicability of four different yield criteria for the finite element modeling of injection-molded low-density polyethylene (LDPE) plates. The yield functions under investigation were von Mises, Hill 1948 from the Hill family and from Hershey’s family, Barlat Yld91, and Barlat Yld2004-18P. These criteria are employed to model the mechanical behavior of LDPE plates under tension, which have been produced via an injection-molding process. To establish the material model, the test results obtained only from simple uniaxial tensile tests in various material orientations were used that can be performed in any material testing lab using a tensile testing machine. This study also used a 2D DIC system for full-field strain measurements, which can also be performed without additional investment using OpenDIC [26], Ncorr [27], or GOM [28] correlate 2D to mention a few.

This paper is structured as follows: Firstly, in Section 2, the material, experimental method, and calibration procedure of the yield criteria are presented. In Section 3, the uniaxial tensile test results, and results from the FE models adopting four yield criteria are presented and compared. Insights into the results of this study are given in this Section and arguments are presented on the accuracy of different yield criteria, followed by some concluding remarks in Section 4. This scientific investigation contributes to the understanding of the mechanical behavior of LDPE plates and provides valuable insights for future research and practical applications of a few anisotropic yield criteria by simplifying the characterization process.

## 2. Materials and Methods

### 2.1. LDPE Material

**Figure 1:**
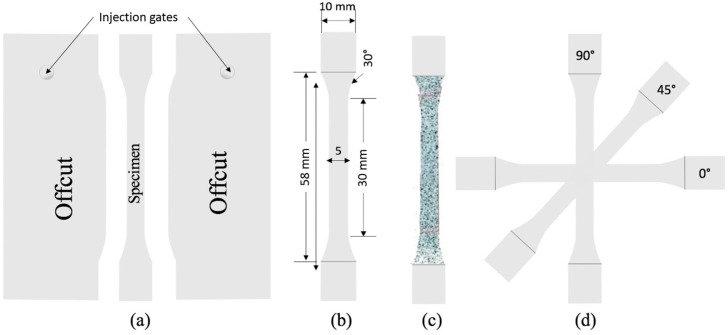
(**a**) Test specimen geometry punched from injection-molded plate, (**b**) specimen dimension between grips (thickness 0.63 mm), (**c**) specimen marked with DIC pattern, and (**d**) a few tested material orientations.

This section provides a concise overview of the preparation of the LDPE plates, test samples, and the experimental methods employed in this study. The dogbone specimen used for testing was cut from low-density polyethylene (LDPE) plates that were manufactured using an injection-molding machine. The LDPE used as the base material was white-pigmented and had a Melt Flow Index (MFI) of approximately 20 g per 10 min (measured at 190 °C and 2.16 kg; following ASTM D1238-04 and ISO 1133:1997 [29]). To produce the polymer testing plates, a horizontal Arburg 470 800-70S hydraulic injection-molding equipment was utilized according to ISO 294-5 [30]. The melted LDPE, with a volume flow rate of 20 cm^3^/s was injected into a tool maintained at a temperature of 40 °C. Subsequently, a cooling process was carried out for 11 s. The production of the testing plates employed a pure injection-molding process, and a floodgate was employed to ensure the uniform evolution of the flow front within the plates. The dogbone test specimens were prepared following the ISO 527-2 1BA [31] standard and were cut using a cutting die and hand press. A sample injection-molded plate, specimen dimensions, and a few material orientations are depicted in Figure 1.

### 2.2. Experimental Methods

In order to gather data on the mechanical behavior of the LDPE plates, tensile tests of dogbone specimens were conducted in seven different material orientations. These orientations were determined by rotating the specimens in 15-degree increments with respect to the molding material flow direction, also known as the MD (as referred to in this article). The other orientations tested were at 15°, 30°, 45°, 60°, 75°, and 90° (CD). The dimensions of the test specimens, depicted in Figure 1c, were measured in millimeters, which had an out-of-plane thickness of 0.63 mm. The specimens were mounted between hydraulic grips and subjected to gradual deformation at a rate of 30 mm/min at room temperature until failure. Throughout the test, the force exerted on the specimens was measured and recorded as a function of global deformation. These data allowed for the acquisition of stress–strain information. To ensure reliable and repeatable results, each specimen was tested three times in its respective material orientation. This approach accounted for the statistical dispersion that may occur in the results [32]. The uniaxial tensile tests were performed in an MTS QTest 100 machine, equipped with a 2 kN load cell, in the laboratory at the BTH (Blekinge Institute of Technology). To capture information about the displacements and strains across the entire specimen surface, acrylic paint was sprayed onto one plane of each specimen, creating a stochastic pattern. The deformations of the specimens were then recorded using a compact 28 mm lens with a 24-megapixel camera, operating at an average magnification of 30 pixels/mm and 120 frames per second. This allowed for full-field in-plane strain measurements using digital image correlation (DIC) analysis performed with GOM correlate software (2018) [28]. The evolution of strains obtained from the DIC was also recorded for later comparison with the finite element (FE) model. These experimental results served as the foundation for the subsequent material calibration and validation of the FE model results. For the identification of the material parameters of the yield criteria and for the FE model input, the following information was collected from the stress–strain response of the material and DIC measurements:

- The uniaxial yield stresses in different material orientations (denoted as *σ*_0_, *σ*_45_, *σ*_90_, etc.);
- The coefficients of uniaxial strain anisotropy (denoted as *r*_0_, *r*_45_, *r*_90_, etc.);
- Hardening stress–strain relation of LDPE in MD.

### 2.3. Anisotropy Parameters

Stain anisotropy is characterized by the Lankford coefficient, *r_θ_*, which is defined as the ratio of the width strain, *ϵ,* to the thickness strain, *ϵ_t_,* increments [13].

(1) $$r_{\theta }=\frac{{\dot{\epsilon }}_{w}}{{\dot{\epsilon }}_{t}}$$

The thickness strain is difficult to accurately measure in a thin sheet, so it is calculated from the longitudinal and width strains by assuming volume conservation. Thus, *ϵ_t_* is defined as in Equation (2) [13].

(2) $$\epsilon_{t}=-(\epsilon_{l}+\epsilon_{w})$$

The in-plane strains, i.e., *ϵ_l_* (length strain) and *ϵ_w_* (width strain), were obtained from experiments using virtual strain gauges in DIC. The values of Poisson’s ratio were also estimated for all seven different material orientations as in Equation (3).

(3) $$\nu_{12}=-\frac{\epsilon_{w}}{\epsilon_{l}}$$

The yield stress ratio (*R_θ_* = *σ_θ_*/*σ*_0_) is the normalized yield stress (i.e., *σ*_0_/*σ*_0_, *σ*_15_/*σ*_0_, *σ*_30_/*σ*_0_, *σ*_45_/*σ*_0_, *σ*_60_/*σ*_0_, *σ*_75_/*σ*_0_, and *σ*_90_/*σ*_0_) taken for all orientations at a 0.23 strain. The motivation for identifying the yield stress at this higher engineering strain of 0.23 instead of the initial yield stress was that the material starts showing a strong anisotropy pattern around this strain in all orientations. As presented later, the studied LDPE shows a hump in force responses at 75° and 90° orientations, which indicates anisotropic hardening. Yield stress is taken after the hump to avoid the overshoot of the force in these two orientations in the finite element simulation. The average values of measured anisotropic parameters are summarized in Table 1.

The R and r values in Table 1 were the basis for yield parameter identification. In this section, the analytical expressions needed and optimization procedures used for material calibration are discussed for different yield criteria.

### 2.4. Anisotropic Yield Criteria Calibration

An elastoplastic material modeling framework was employed to capture the in-plane material anisotropy observed in the injection-molded LDPE plates. The elastic material response was assumed to be linear and isotropic. This assumption is backed by the experimental stress–strain relations in different orientations and earlier studies on the same grade of LDPE [1,18], all showing a small magnitude of initial yield stress compared with the maximum stress and relatively small spread of Young’s modulus in the different orientations presented in Table 1. Three crucial components are utilized to describe plastic material behavior under general stress states. The first component is a yield criterion that expresses the relationship between the stress components. This criterion plays a vital role in determining the initiation of plastic deformation. The second component is a flow rule, which establishes the relationship between the components of the strain rate and stress. This rule governs the plastic deformation process. Lastly, a hardening rule is employed to describe the evolution of the initial yield stress during the material’s deformation process. In the following subsections, the calibration procedure of different yield criteria employed in this study is described. These calibrated constitutive parameters facilitate FE modeling and analysis.

#### 2.4.1. von Mises

Among the various isotropic plasticity models available, the von Mises yield function [33] is one of the most commonly used [34]. Interestingly, the calibration of this comprehensive model requires only uniaxial tensile test data, eliminating the need for extensive material characterization procedures. The von Mises yield function can be represented by six stress components in three dimensions, as defined in Equation (4):

(4) $$f\left(\sigma_{ij}\right)={\left(\sigma_{11}-\sigma_{22}\right)}^{2}+{\left(\sigma_{22}-\sigma_{33}\right)}^{2}+{\left(\sigma_{33}-\sigma_{11}\right)}^{2}+6\sigma_{23}^{2}+6\sigma_{31}^{2}+6\sigma_{12}^{2}=2{\underline{\sigma }}^{2}$$

This equation effectively captures the relationship between the stress components and enables the determination of the yield point.

#### 2.4.2. Hill48

In 1948, Hill, R. [6] proposed this anisotropic yield criterion as a generalization of the Huber–Mises–Hencky criterion. The yield criterion is expressed with the quadratic function described in Equation (5). Indices 1, 2, and 3 as subscripts express machine, transverse in-plane, and out-of-plane orientations. The planar anisotropy of materials is described with the six material parameters F, G, H, L, M, and N.

(5) $$2f\left(\sigma_{ij}\right)=F{\left(\sigma_{22}-\sigma_{33}\right)}^{2}+G{\left(\sigma_{33}-\sigma_{11}\right)}^{2}+H{\left(\sigma_{11}-\sigma_{22}\right)}^{2}+2L\sigma_{23}^{2}+2M\sigma_{31}^{2}+2N\sigma_{12}^{2}=1$$

These parameters for the studied LDPE were calibrated by optimizing the cost function in Equation (6).

(6) $$Cost\left(F,G,H,N\right)={\left(\frac{R_{0{}^{\circ}}^{pr}}{R_{0{}^{\circ}}^{exp}}-1\right)}^{2}+{\left(\frac{R_{45{}^{\circ}}^{pr}}{R_{45{}^{\circ}}^{exp}}-1\right)}^{2}+{\left(\frac{R_{90{}^{\circ}}^{pr}}{R_{90{}^{\circ}}^{exp}}-1\right)}^{2}$$

(7) $$R_{\theta {}^{\circ}}^{pr}\left(\theta ,\;F,\;G,\;H,\;N\right)=\frac{1}{Fsin^{4}\theta +Gcos^{4}\theta +Hcos^{2}2\theta +2Nsin^{2}\theta cos^{2}\theta }$$

The expressions $R_{\theta {}^{\circ}}^{exp}$ and $R_{\theta {}^{\circ}}^{pr}$ in Equation (6) are the experimental and theoretical (predictor) stress anisotropic ratio at a  θ° material orientation, respectively. The $R_{\theta {}^{\circ}}^{exp}$ values were found from the stress–strain curve of a tensile test at a *θ*° (0°, 45°, and 90°) material orientation, and $R_{\theta {}^{\circ}}^{pr}$ were calculated using Equation (7).

The MATLAB optimization function ‘fmincon’ was used with specific starting points, [0.5 0.5 0.5 0.5], lower limits [0.25 0.25 0.25 0.25], and upper limits [2 2 2 2] of parameters F, G, H, and N, respectively. The values were systematically changed until the code found the optimum minimum value of the cost function. The remaining Hill48 parameters were set to be one, i.e., L = M = 1. The optimum values for F, G, H, and N are shown in Table 2.

These parameters were then used to find the plastic coefficients using Equations (8)–(16), and the values of the plastic coefficients are presented in Table 3.

(8) $$F=\frac{1}{2}\left(\frac{1}{{R_{22}}^{2}}+\frac{1}{{R_{33}}^{2}}-\frac{1}{{R_{11}}^{2}}\right)$$

(9) $$G=\frac{1}{2}(\frac{1}{{R_{11}}^{2}}+\frac{1}{{R_{33}}^{2}}-\frac{1}{{R_{22}}^{2}})$$

(10) $$H=\frac{1}{2}(\frac{1}{{R_{11}}^{2}}+\frac{1}{{R_{22}}^{2}}-\frac{1}{{R_{33}}^{2}})$$

(11) $$\;L=\frac{3}{{{2R}_{23}}^{2}}$$

(12) $$R_{11}=\sqrt{\frac{1}{G+H}}$$

(13) $$R_{22}=\sqrt{\frac{1}{F+H}}$$

(14) $$R_{33}=\sqrt{\frac{1}{G+F}}$$

(15) $$R_{33}=\sqrt{\frac{3}{2N}}$$

(16) $$R_{13}=R_{23}=1\;$$

#### 2.4.3. Barlat2004-18P

Barlat et al. proposed the Yld2004-18P yield criterion, which describes the anisotropic behavior of materials in a full three-dimensional stress state and is expressed with Equation (17) [10].

(17) $$\;\phi \left(\tilde{S^{\prime }},\tilde{S^{\prime \prime }}\right)={\left|\tilde{S_{1}^{\prime }}-\tilde{S_{1}^{\prime \prime }}\right|}^{a}+{\left|\tilde{S_{1}^{\prime }}-\tilde{S_{2}^{\prime \prime }}\right|}^{a}+{\left|\tilde{S_{1}^{\prime }}-\tilde{S_{3}^{\prime \prime }}\right|}^{a}+{\left|\tilde{S_{2}^{\prime }}-\tilde{S_{1}^{\prime \prime }}\right|}^{a}+{\left|\tilde{S_{2}^{\prime }}-\tilde{S_{2}^{\prime \prime }}\right|}^{a}+{\left|\tilde{S_{2}^{\prime }}-\tilde{S_{3}^{\prime \prime }}\right|}^{a}+{\left|\tilde{S_{3}^{\prime }}-\tilde{S_{1}^{\prime \prime }}\right|}^{a}+{\left|\tilde{S_{3}^{\prime }}-\tilde{S_{2}^{\prime \prime }}\right|}^{a}+{\left|\tilde{S_{3}^{\prime }}-\tilde{S_{3}^{\prime \prime }}\right|}^{a}=4{\underline{\sigma }}^{a}$$

**Figure 2:**
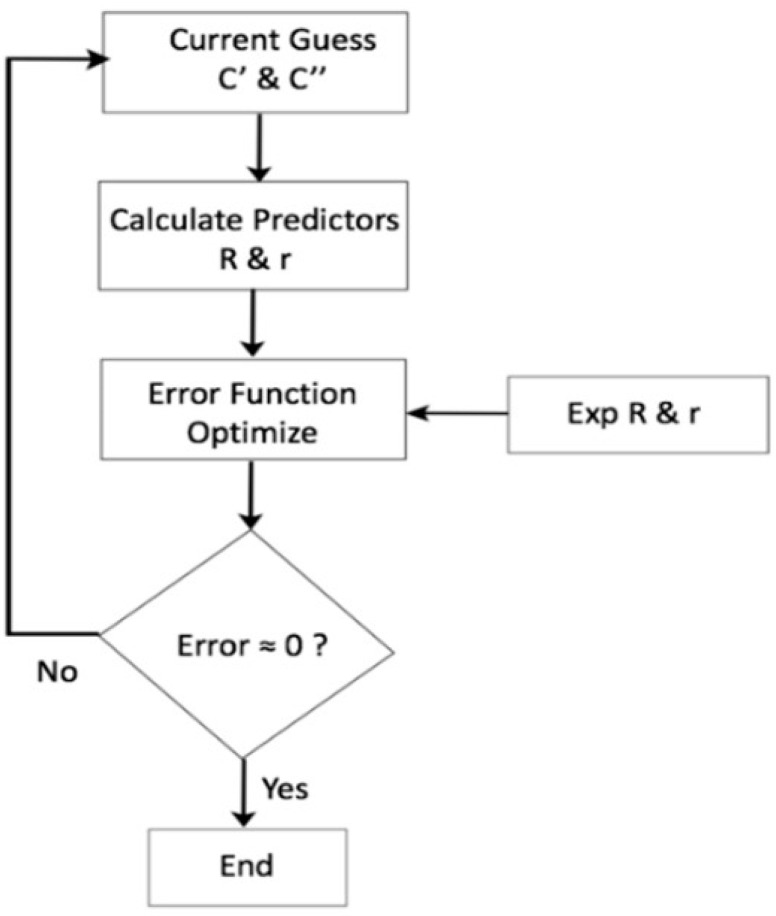
Flowchart for Yld2004-18P plastic coefficient optimization algorithm.

This yield function contains 18 parameters, as indicated by -18P in the name. Barlat Yld2004-18P can be used when several experimental data are available, such as the uniaxial tension data in seven test orientations between MD and CD, as well as the balanced biaxial teat data. Four additional out-of-plane shear tests are needed for a comprehensive calibration of the Barlat Yld2004-18P model. There is no simple experiment to measure a balanced biaxial stress–strain response, and the out-of-plane shear stress components were difficult to measure as the LDPE plate material was 0.63 mm thick. In this study, only seven uniaxial test information (Yield stress and r) were used, and an optimization procedure was forced to find 18 optimum plastic coefficients (*c_ij_*′, *c_ij_*″) for the model. It is also possible to theoretically predict biaxial yield stress and strain anisotropy using Hill48 or Barlat Yld89 prediction, for example. This is a significant deviation from the standard calibration procedure. However, if this advanced model can be calibrated with only the simple tensile tests with some trade-offs in accuracy, this can open the possibility of adopting this model in a wide number of numerical studies. The 18 coefficients of the Barlat Yld2004-18P model were identified using the optimization scheme in Figure 2. E (*c_ij_*′, *c_ij_*″) in Equation (18) is the cost function (error), which is a function of 18 plastic coefficients. The expressions $r_{q}^{exp}$ and $r_{q}^{pr}$ are the experimental and theoretical (predictor) strain anisotropic ratio and, $\sigma_{p}^{exp}$ and $\sigma_{p}^{pr}$ are experimental and theoretical yield stress in a *_θ_*° material orientation. The exponent ‘a’ as in Equation (17) was assumed in this study to be 8 based on a pre-study of LDPE for this article [18].

(18) $$E\left(c_{ij}^{\prime },c_{ij}^{\prime \prime }\right)=\sum_{p}w_{p}{\left(\frac{\sigma_{p}^{pr}}{\sigma_{p}^{exp}}-1\right)}^{2}+\sum_{q}w_{q}{\left(\frac{r_{q}^{pr}}{r_{q}^{exp}}-1\right)}^{2}$$

*w_p_* is the weight related to the yield stress taking a value of 10, and *w_q_* is the weight related to the anisotropic ratio (r) taking a value of 1 in this study. The higher weight associated with the yield stress prioritizes its optimization over r. The predictors $\sigma_{p}^{pr}$ and $r_{q}^{pr}$ are calculated according to Equations (19)–(30).

(19) $$\frac{\sigma_{at\;\theta }^{pr}}{\bar{\sigma }}={\left(\frac{4}{\phi \left(s_{\theta }\right)}\right)}^{1/a}$$

(20) $$r_{at\;\theta }^{pr}=\frac{\frac{\partial \phi }{\partial \sigma_{11}}sin^{2}\theta +\frac{\partial \phi }{\partial \sigma_{22}}cos^{2}\theta -2\frac{\partial \phi }{\partial \sigma_{12}}cos\theta sin\theta }{\left(\frac{\partial \phi }{\partial \sigma_{11}}+\frac{\partial \phi }{\partial \sigma_{22}}\right)}$$

(21) $$s_{\theta }=\left[\left({cos}^{2}\theta -\frac{1}{3}\right),\;\;\;\;\;\left({sin}^{2}\theta -\frac{1}{3}\right),\;\;\;-\frac{1}{3},\;\;\;0,\;\;\;0,\;\;\;sin\theta cos\theta \right]$$

To express the yield function $\phi \left(\tilde{S^{\prime }},\tilde{S^{\prime \prime }}\right)$, the principal values $\tilde{S^{\prime }},\tilde{S^{\prime \prime }}$ are obtained as in Equations (22)–(24).

(22) $$\tilde{S_{1}}=2\sqrt{H_{1}^{2}+H_{2}}cos\left(\frac{\theta }{3}\right)+H_{1}$$

(23) $$\;\tilde{S_{2}}=2\sqrt{H_{1}^{2}+H_{2}}cos\left(\frac{\theta +4\pi }{3}\right)+H_{1}$$

(24) $$\tilde{S_{3}}=2\sqrt{H_{1}^{2}+H_{2}}cos\left(\frac{\theta +2\pi }{3}\right)+H_{1}$$

where

(25) $$H_{1}=\left({\tilde{s}}_{11}+{\tilde{s}}_{22}+{\tilde{s}}_{33}\right)/3$$

(26) $$H_{2}=\left({\tilde{s}}_{23}^{2}+{\tilde{s}}_{31}^{2}+{\tilde{s}}_{12}^{2}-{\tilde{s}}_{22}{\tilde{s}}_{33}-{\tilde{s}}_{33}{\tilde{s}}_{11}-{\tilde{s}}_{11}{\tilde{s}}_{22}\right)/3$$

(27) $$H_{3}=\left(2{\tilde{s}}_{23}{\tilde{s}}_{31}{\tilde{s}}_{12}+{\tilde{s}}_{11}{\tilde{s}}_{22}{\tilde{s}}_{33}-{\tilde{s}}_{11}{\tilde{s}}_{23}^{2}-{\tilde{s}}_{22}{\tilde{s}}_{31}^{2}-{\tilde{s}}_{33}{\tilde{s}}_{12}^{2}\right)/2$$

where the tensor $\tilde{s}\left({\tilde{s}}^{\prime }\;and\;\;{\tilde{s}}^{\prime \prime }\right)$ is represented in Equation (28).

(28) $$\;\tilde{s}=\left[\begin{array}{ccc} {\tilde{s}}_{11} & {\tilde{s}}_{12} & {\tilde{s}}_{13}\\ {\tilde{s}}_{21} & {\tilde{s}}_{22} & {\tilde{s}}_{23}\\ {\tilde{s}}_{31} & {\tilde{s}}_{32} & {\tilde{s}}_{33} \end{array}\right]$$

Furthermore, *σ* is the Cauchy stress tensor, and ${\tilde{s}}^{\prime }=C\prime T\sigma$, ${\tilde{s}}^{\prime \prime }=C^{\prime \prime }T\sigma$, where *C* (*C*′ and *C*″) are the matrix containing the coefficients.

(29) $$\;C=\left[\begin{array}{cc} \begin{array}{ccc} 0 & {-c}_{12} & {-c}_{13}\\ {-c}_{21} & 0 & {-c}_{23}\\ {-c}_{31} & {-c}_{32} & 0 \end{array} & \begin{array}{ccc} 0 & \;\;\;0 & \;\;\;\;0\\ 0 & \;\;\;0 & \;\;\;\;0\\ 0 & \;\;\;0 & \;\;\;\;0 \end{array}\\ \begin{array}{ccc} 0 & \;\;\;\;0 & \;\;\;\;\;0\\ 0 & \;\;\;\;0 & \;\;\;\;\;0\\ 0 & \;\;\;\;0 & \;\;\;\;\;0 \end{array} & \begin{array}{ccc} c_{44} & 0 & 0\\ 0 & c_{55} & 0\\ 0 & 0 & c_{66} \end{array} \end{array}\right]$$

(30) $$\;T=\frac{1}{3}\left[\begin{array}{cc} \begin{array}{ccc} 2 & -1 & -1\\ -1 & 2 & -1\\ -1 & -1 & 2 \end{array} & \begin{array}{ccc} 0 & 0 & 0\\ 0 & 0 & 0\\ 0 & 0 & 0 \end{array}\\ \begin{array}{ccc} 0 & \;0 & \;\;0\\ 0 & \;0 & \;\;\;0\\ 0 & \;0 & \;\;\;0 \end{array} & \begin{array}{ccc} 3 & 0 & 0\\ 0 & 3 & 0\\ 0 & 0 & 3 \end{array} \end{array}\right]$$

The output of the optimization was 18 parameter values for defining the constitutive of Barlat Yld2004-18P. As only 14 measured quantities in terms of different yield stress and r values were used to optimize 18 parameters, there was a risk of attaining a local optimum, leading to an impractical simulation response when used with the FE solver. A Robust MATLAB built-in optimization toolbox ’fmincon’ was used with the ‘interior-point’ algorithm. The convergence tolerance was set to a low value of 1 × 10^−24^. Another major step to avoid reaching the local optimum was to define closer-to-optimal initial values of the coefficient and use a small but enclosing range of each coefficient to search for the optimum. In this study, with the exponent ‘a’ value being 8, the initial value, lower bound, and upper bound of all parameters were 0.5, −2, and 2, respectively. This will be the recommendation to use with polyethylene polymers. The flowchart for the Yld2004-18P plastic coefficient optimization is depicted in Figure 2.

#### 2.4.4. Barlat Yld91

Barlat Yld91 is a general six-component yield criterion for anisotropic materials. Six coefficients, a, b, c, f, g, and h, describe the anisotropy of the material with a chosen exponent. They are identified via three uniaxial tensile tests in the directions of the orthotropic axes (a, b, and c) and three pure shearing tests (f, g, and h). However, in this study, as proposed by Barlat, F. et al., Barlat2004-18P was reduced to Barlat Yld91 by setting the plastic coefficients of the 1st transformation (C′) equal to those of the 2nd transformation (C″) and setting several constraints among a few of the coefficients [10]. The constraints were as in in Equations (31)–(33).

(31) $$3c_{13}^{\prime }=2c_{31}^{\prime }+2c_{12}^{\prime }-c_{23}^{\prime }$$

(32) $$3c_{32}^{\prime }=2c_{23}^{\prime }+2c_{31}^{\prime }-c_{12}^{\prime }$$

(33) $$3c_{21}^{\prime }=2c_{12}^{\prime }+2c_{23}^{\prime }-c_{31}^{\prime }$$

Finally, the plastic coefficients were:

(34) $$a=\left(4c_{23}^{\prime }+c_{31}^{\prime }-2c_{12}^{\prime }\right)/3$$

(35) $$b=\left(4c_{31}^{\prime }+c_{12}^{\prime }-2c_{23}^{\prime }\right)/3$$

(36) $$c=\left(4c_{12}^{\prime }+c_{23}^{\prime }-2c_{31}^{\prime }\right)/3$$

(37) $$f=c_{44}^{\prime },\;g=c_{55}^{\prime },\;h=c_{66}^{\prime }$$

To find the LDPE Barlat Yld91 coefficients of LDPE, the yield stress and anisotropic ratio r for three different material orientations (0°, 45°, and 90°) were used in the same optimization code, as used in Barlat Yld2004-18P, together with the constraints imposed for the model as described above. The optimization was sub-optimal as no shear test results were used. To improve this drawback, similar measures were taken as before to avoid local optima of the identified coefficients. The material parameters are given in the Table 4.

**Figure 3:**
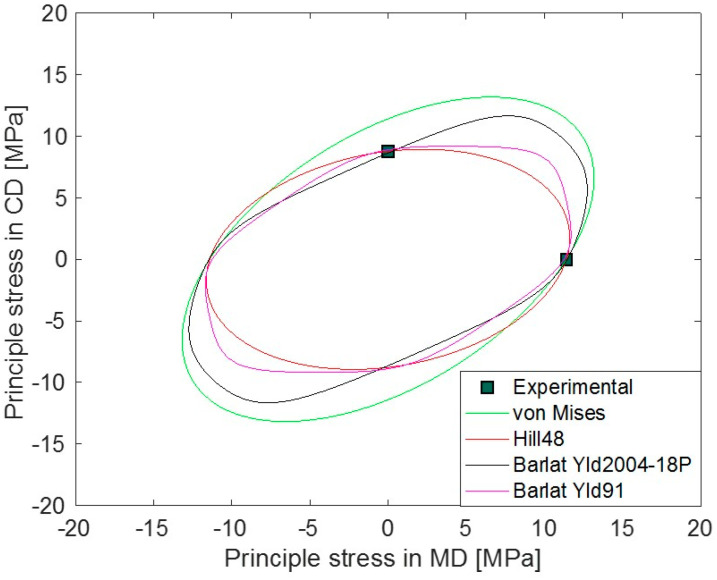
Yield surfaces in principal stress plane for Hill48, von Mises, Barlat Yld91. and Barlat Yld2004-18P.

All the calibrated initial yield surfaces are visualized in the principal stress plane together with the experimental measurements in the MD and CD in Figure 3. Except for von Mises, other anisotropic yield criteria can identify differences in MD and CD yield stresses.

### 2.5. Isotropic Hardening

Issotropic hardening demonstrates a gradual increase in yield stress as plastic strain increases. In other words, the yield surface stays centered at the origin and grows any time plastic deformation takes place. The simplest way of characterizing the true stress–strain relation of the isotropic hardening curve is from the uniaxial tensile test. As the name suggests, this relation is considered in only one material orientation, in this article, that was MD.

**Figure 4:**
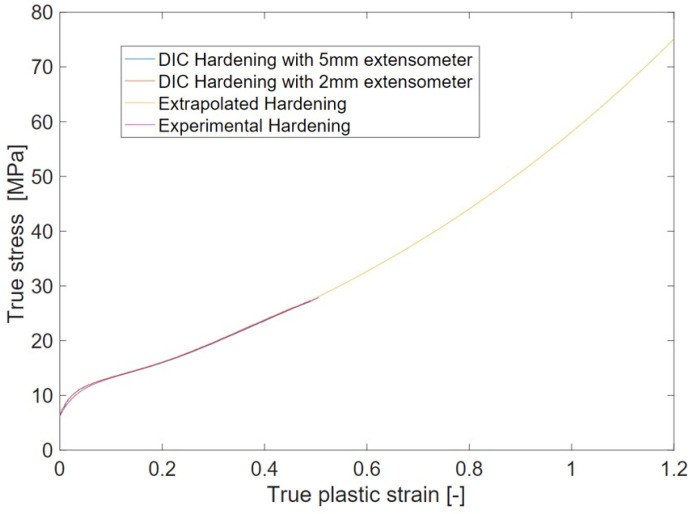
Experimental, DIC, and extrapolated hardening curve in MD of LDPE.

From the global force and displacement measurements of a tensile test, the hardening curve could be derived only up to a plastic strain of 0.5. However, the LDPE plate underwent much higher strain before strain localization. An attempt to capture more local stress–strain relations by using DIC measurements only minorly improved the measured plastic strain limit. To overcome this limitation, the hardening curve was extrapolated using the Swift/Hockett–Sherby law described by Equation (38) and shown in Figure 4. The constants in Equation (38), i.e., *C_i_* and *α,* were determined by fitting the experimental true stress and true plastic strain curve in the MD.

(38) $$\sigma_{\mathrm{SHS}}=\left(1\;-\;\alpha \right)C_{1}{\left(C_{2}+\varepsilon \right)}^{C_{3}}+\alpha \left(C_{2}\;-\;\left(C_{2}\;-\;C_{1}\right)e^{\left(-C_{3}\varepsilon^{C_{4}}\right)}\right)$$

### 2.6. FE Modeling

**Figure 5:**
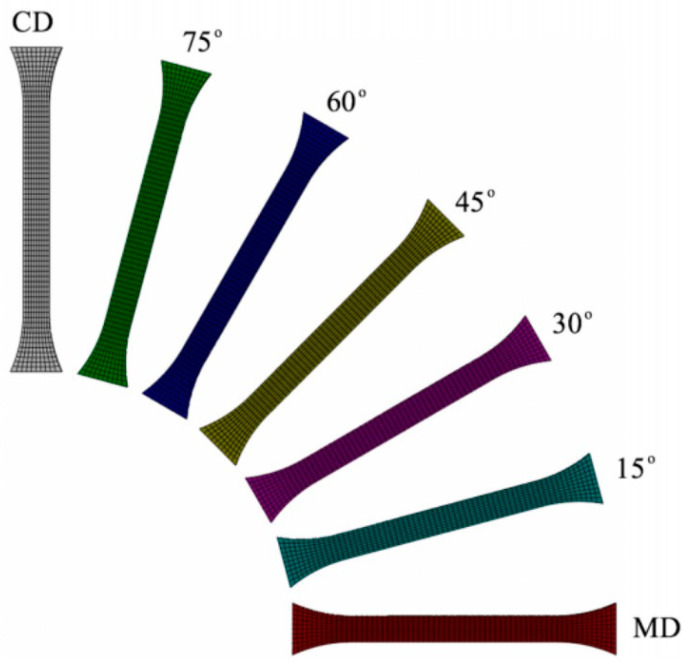
Undeformed FE mesh in the seven different material orientations.

In the finite element (FE) model, the same dimensions and boundary conditions as the experimental dogbone test specimens were employed. Considering the capabilities of the Abaqus™ R2020 software, the Explicit solver was selected for simulation with all the yield criteria utilized in this study, i.e., von Mises, Hill48, Barlat Yld2004-18P, and Barlat Yld91. Assuming a linear, isotropic elastic deformation, an MD Young’s modulus of 240 MPa and MD Poisson’s ratio of 0.37 were used. The material density was 9 × 10^−10^ ton/mm^3^. The calibrated plastic coefficients for the different yield criteria were employed in the model. To describe the stress–strain response, the identified isotropic hardening curve in the MD was used. The simulations were conducted with the nonlinear geometry turned on to account for the large deformations. A C3D8R 8-node linear brick element with reduced integration was utilized in the Abaqus explicit solver. The element size was approximately 1 mm in the length and width directions, and 0.63 mm in the thickness direction. The undeformed mesh of specimens in the seven different material orientations is shown in Figure 5. It can be seen that the tension in the dogbone specimens in all 7 orientations was simulated in a single model. Although an overall mass scaling of 100 was used to reduce the computation time, it was checked that the kinetic energy in the model was less than 0.01% of the internal and total energy of the model and did not significantly affect the solution.

## 3. Results and Discussion

### 3.1. Experimental Results

**Figure 6:**
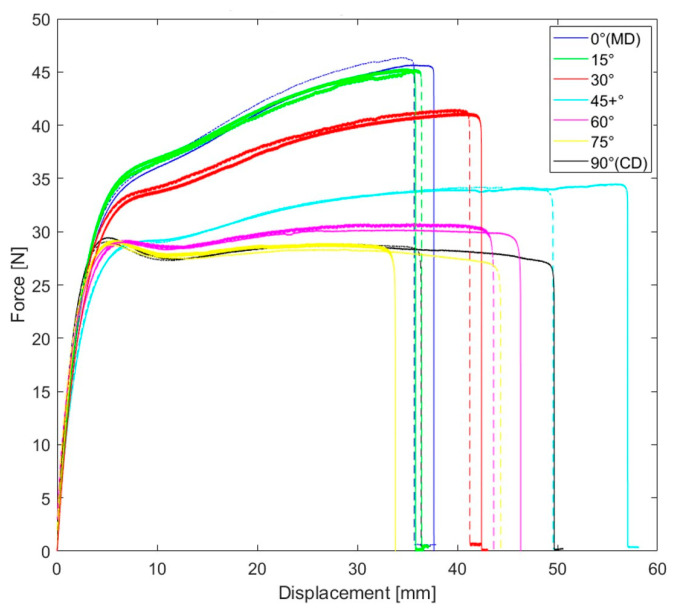
Experimental force–displacement response for seven different material orientations.

**Figure 7:**
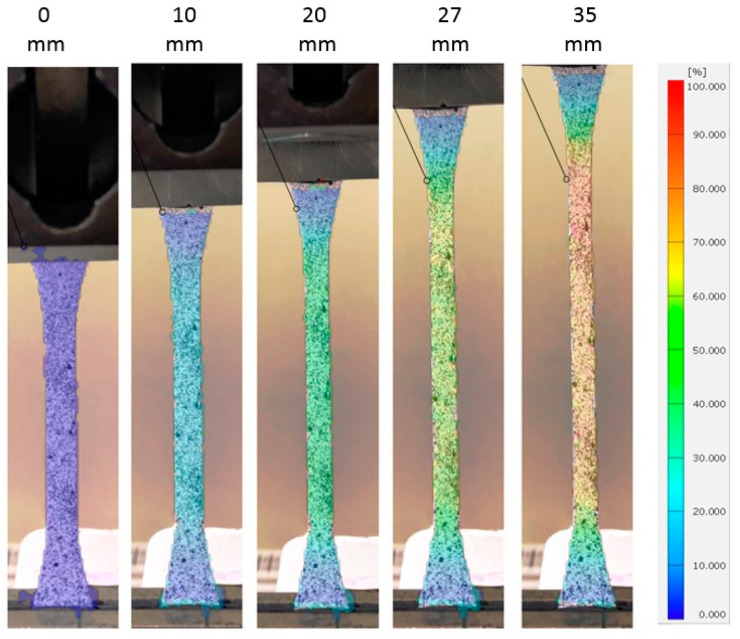
Maximum principal strain distribution at different deformation sequences in MD orientation.

Figure 6 presents the representative force–displacement responses observed in each material orientation, offering a visual of some mechanical characteristics of the LDPE plates. It is clear that the studied LDPE is highly anisotropic in hardening and failure. These curves were the basis for the yield stress anisotropic ratio and hardening curve characterization. It can also be concluded that the tests were repeatable. Furthermore, Figure 7 illustrates the measured full-field maximum principal strain evolution during a tensile test in MD. The Young’s modulus, Poisson’s ratio, and anisotropic ratio (r) were calculated based on these strain measurements. The average values of these material properties are reported in Table 1.

### 3.2. FE Simulation Results

**Figure 8:**
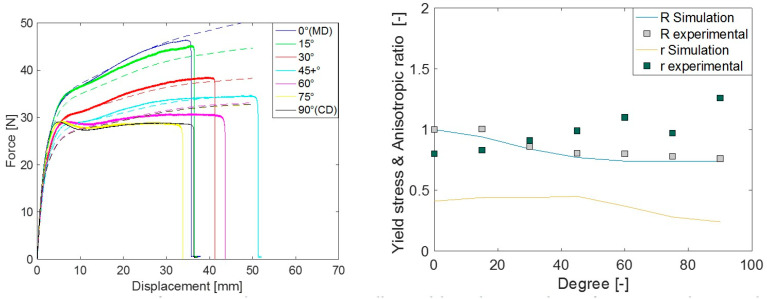
Comparison of experimental measurements vs. Hill48 model simulation prediction for force-displacement (**left**) and yield stress ratio ‘R’ and anisotropic ratio ‘r’ (**right**).

**Figure 9:**
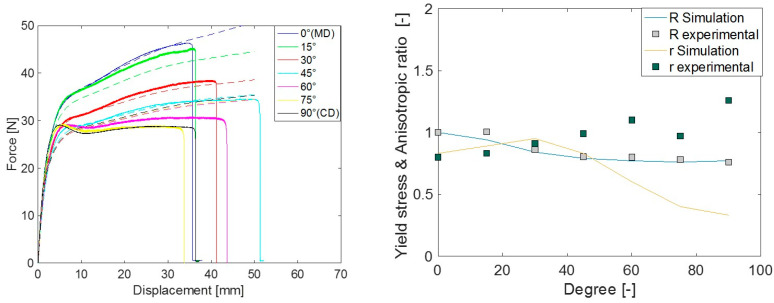
Comparison of experimental measurement vs. Barlat Yld91 model simulation prediction for force-displacement (**left**) and yield stress ratio ‘R’ and anisotropic ratio ‘r’ (**right**).

**Figure 10:**
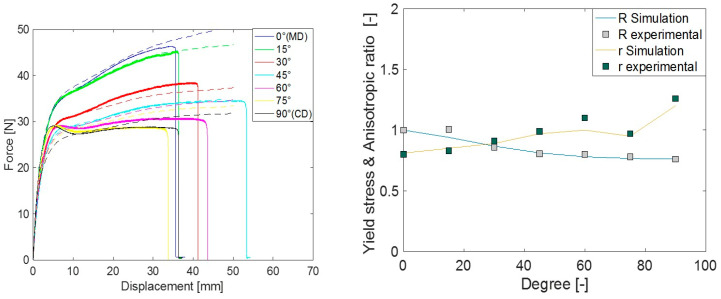
Comparison of experimental measurement vs. Barlat Yld2004-18P model simulation prediction for force-displacement (**left**) and yield stress ratio ’R’ and anisotropic ratio ’r’ (**right**).

**Figure 11:**
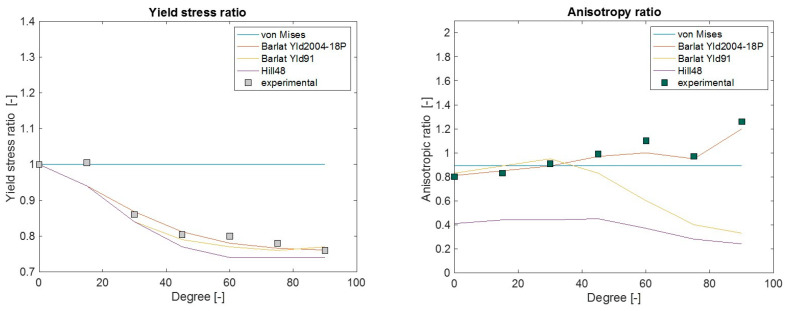
Comparison of experimental measurement vs. FE simulation prediction for yield stress ratio ‘R’ (**left**) and anisotropic ratio ‘r’ (**right**) using different yield criteria.

The force–displacement response, anisotropic ratio (r), and yield stress ratio (R) response from the simulation using different yield criteria were evaluated in different material orientations. Comparative plots of these quantities with the experimental measurements are presented in Figure 8, Figure 9 and Figure 10. Both experimental and simulated R and r values evolved with the level of plastic strain, so their comparison was made at an engineering strain of 0.23. The comparisons of r and R prediction goodness between all the yield criteria used are depicted in Figure 11.

In general, when a yield criterion is calibrated against experimental R values, the force–displacement response of the simulation approaches that of the experiment. As the Hill48 parameters were calibrated against R (in 0°, 45°, and 90°), the simulation R as well as force–displacement (F-D) response was closer in those orientations, except in 90°. This was a common limitation in all studied yield criteria as there is a distinct hump in the force response at around 5 mm deformation in orientations between 60° to 90°. As isotropic hardening was considered in all simulations, the choice of yield criteria alone could not improve the F-D prediction in these orientations. The simulated r predictions of Hill48 were poor. This means the in-plane deformation and thinning of the material were not captured accurately as these deformations are governed by the parameter r. For Barlat Yld91 calibration, both R and r in three material orientations (0°, 45°, and 90°) were used, and as a result, the F-D, R, and r simulation responses more closely agreed compared to Hill48. It should be noted that although the r value in 90° was used for the Barlat Yld91 plastic coefficient calibration, the experimental and simulated r in 90° was far off. This may be due to the optimization procedure getting trapped in a local optimum. It is recommended to carry out a similar check when this simplified calibration technique only based on tensile tests is performed. Many times, the R and r values in the 0° to 90° orientations were roughly the interpolation of values in 0°, 45° and 90°. In those cases, Barlat Yld91 is an even more robust model for anisotropic simulations.

To model anisotropy in LDPE using only tensile test data, the most accurate yield criterion studied in this article was Barlat Yld2004-18P. This can be seen in the comparisons of R, r, and F–D in Figure 10. The above comparisons investigated the yield criteria accuracy in the F–D, R, and r matrix. Different yield criteria inhabit different levels of flexibility in capturing these responses from the experiments. The more experimental values (R and r) a yield criterion requires for calibration, the more accurate the simulated prediction.

**Figure 12:**
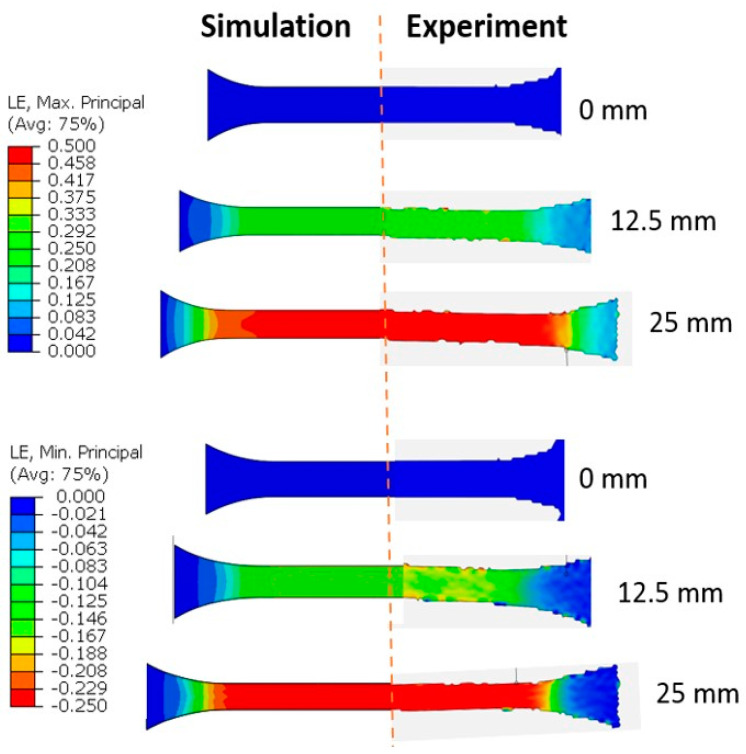
Comparison of true major strain (**top**) and minor strain (**bottom**) distribution obtained from DIC and Yld2004- 18P model simulation at three deformation levels.

Another accuracy check was possible by comparing the simulation and experimental full-field strain evolution with increased deformation. When the principal major and minor strain fields from the DIC and from Barlat Yld2004-18P prediction were compared at three different levels of deformation, the simulation strain field predictions were very accurate even at a higher strain (see Figure 12). The simulation strain fields are expected to agree with the experiment when a yield criterion satisfies the measured r values, which was the case for Barlat Yld2004-18P. Similarly, for the other yield criteria, the strain field was observed to be as accurate as their simulation r prediction shown in Figure 11.

A comparison of the parameter calibration time and simulation CPU (four processors) time in Abaqus 2020 when using different yield criteria is presented in Table 5. The CPU time with Barlat Yld2004-18P was impressively lower than with Barlat Yld91 in Abaqus2020 and not significantly higher than with von Mises and Hill48. This makes Barlat Yld2004-18P a more attractive choice. The optimization time is less significant as this is performed only once for a material.

**Figure 13:**
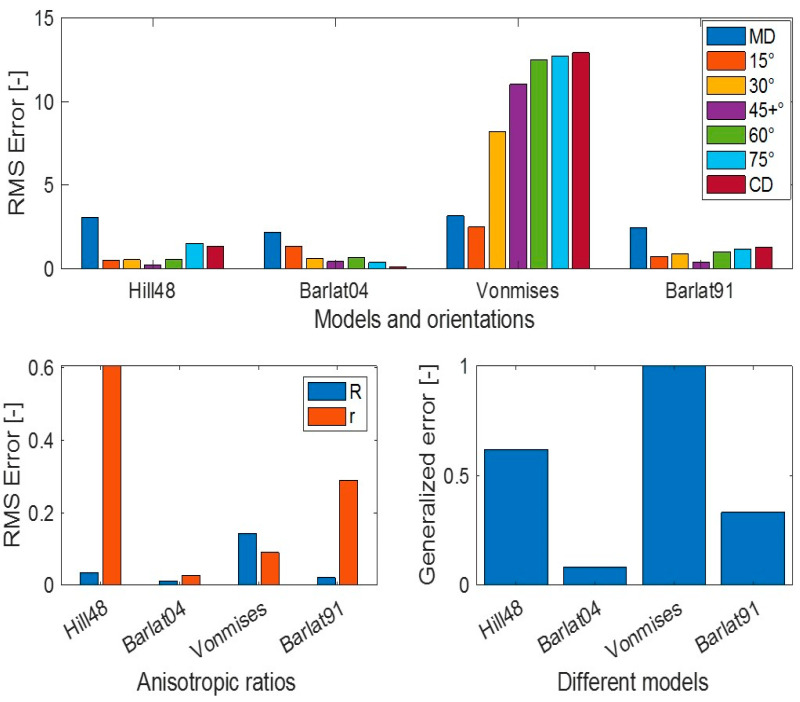
RMS error analysis in different yield criteria.

For a quick check of different yield criteria’s accuracy, the simulation prediction error in F-D, R, and r in all orientations are generalized to a single quantity according to Equation (39), which is called “generalized error” in Figure 13. In this expression, w is the weight factor, which was one in this study, but can be adjusted based on priority. Figure 13 indicates that a more advanced Barlat Yld2004-18P should clearly be prioritized if higher accuracy is desired. Hill48 and Barlat Yld91 have similar accuracy in F-D prediction, but Barlat Yld91 performs better in r prediction, which is very significant if simulation deformations in the width and thickness of the plate are compared with those in the physical experiment.

(39) $$\varepsilon_{model}^{g}=w_{FD}\sum_{\theta =0{}^{\circ}}^{\theta =90{}^{\circ}}\frac{\varepsilon_{FD}}{max(\varepsilon_{FD})}+w_{R}\sum_{\theta =0{}^{\circ}}^{\theta =90{}^{\circ}}\frac{\varepsilon_{R}}{max(\varepsilon_{R})}+w_{r}\sum_{\theta =0{}^{\circ}}^{\theta =90{}^{\circ}}\frac{\varepsilon_{r}}{max(\varepsilon_{r})}$$

### 3.3. Validation of Barlat Yld2004-18P in a Nonstandard Tensile Test Model

**Figure 14:**
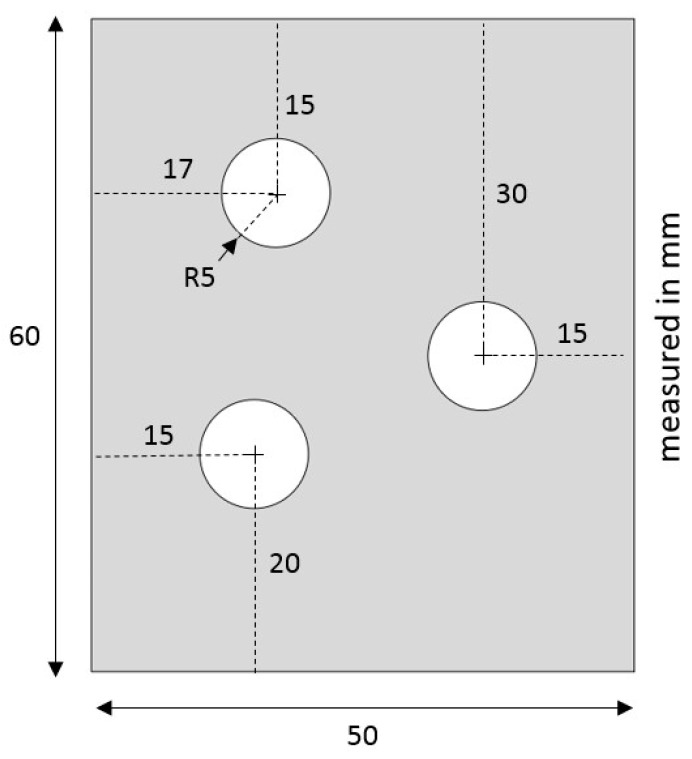
Nonstandard open-hole tensile test specimen.

The accuracy of Barlat Yld2004-18P in particular was tested in the modeling of a nonstandard tensile test specimen from a LDPE plate with three asymmetric open holes. The geometry of the specimen is shown in Figure 14.

**Figure 15:**
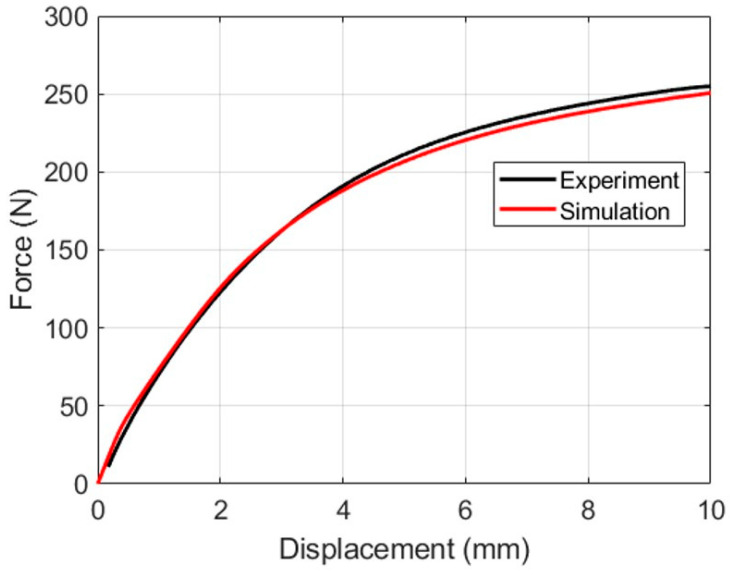
Comparison of experimental and simulated force–displacement of the nonstandard open-hole tensile test.

**Figure 16:**
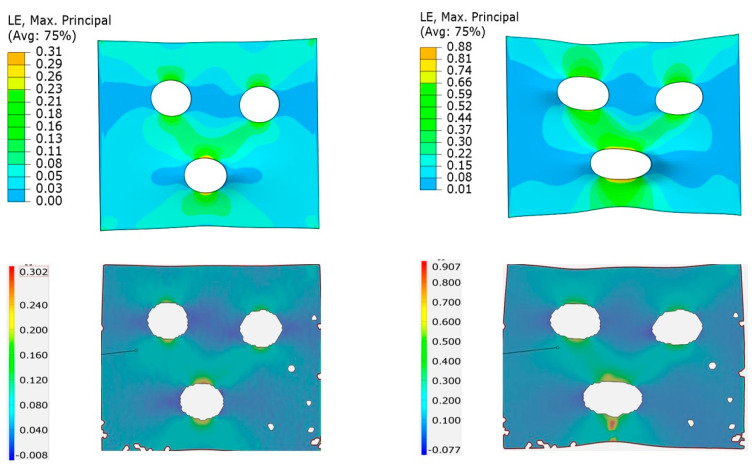
Comparison of experimental (**bottom**) and simulated (**top**) maximum principal strain field of the non-standard open-hole tensile test at two different strain levels.

The specimen was designed such that the stress and strain field as well as stress-triaxiality were nonhomogeneous. The specimens were cut from the same LDPE injection-molded plate set, as used in the dogbone. The holes were cut using a punch-and-die setup with a clearance of 20% of the plate thickness. Furthermore, the designed specimen was tested and the experimental force–displacement and full-field strain were recorded. This experiment was modeled in Abaqus with the Barlat Yld2004-18P yield criteria. The force–displacement comparison between the experiment and simulation shows good agreement in Figure 15. Moreover, the maximum principal strain fields were also compared at two different strain levels in Figure 16. These comparisons strengthen the claim of higher FE modeling accuracy achieved by adopting the Barlat Yld2004-18P yield criteria, even with the simplified characterization routine presented in this work.

## 4. Conclusions

Several elastoplastic anisotropic yield criteria were investigated to simulate high anisotropic response in an injection-molded LDPE plate that can undergo very large deformation before strain localization. The simulation results were compared with physical experiments, and Barlat Yld2004-18P is the recommended yield criterion for FE modeling polyethylene based on accuracy and computation time. A major contribution of this article was the successful calibration procedure of two Barlat anisotropic yield criteria of different complexities only by using uniaxial tensile test data. This simplified calibration procedure proved to bring greater accuracy in anisotropy model prediction at a low cost. However, with a reduced number of experimental data, the characterized yield parameters may be suboptimal, and this study provided a few recommendations to improve the accuracy. 

## Figures and Tables

**Table 1:** Young’s modulus, yield stress ratios, anisotropic ratios, and Poisson’s ratios computed from tensile tests in seven different material orientations.

Orientation (*θ*)	Yield Stress Ratio (R)	Anisotropic Ratio (r)	Poisson’s Ratio (*ν*)	Young’s Modulus (MPa)
0°	1.00	0.80	0.37	240 ± 1
15°	1.01 ± 0.01	0.83	0.39	281 ± 2
30°	0.86 ± 0.02	0.91	0.40	260 ± 2
45°	0.80 ± 0.005	0.99	0.40	203 ± 2
60°	0.80 ± 0.01	1.10	0.46	190 ± 2
75°	0.78 ± 0.005	0.97	0.39	250 ± 1
90°	0.76 ± 0.005	1.26	0.50	232 ± 1

**Table 2:** Hill48 yield coefficients.

F	G	H	L	M	N
1.44	0.75	0.25	1.00	1.00	1.99

**Table 3:** Hill48 plastic coefficients for LDPE to be used in Abaqus.

*R*11	*R*22	*R*33	*R*12	*R*13	*R*23
1.00	0.77	0.68	0.87	1.00	1.00

**Table 4:** Plastic coefficients for Barlat Yld91 optimized with three different material orientations (0°, 45°, and 90°).

a	b	c	f	g	h
1.52	1.02	1.00	0.97	0.97	1.25

**Table 5:** CPU times for simulations (dogbone models) and parameter identification run times for different anisotropic yield criteria in seconds.

Yield Function	CPU Time (s)	Optimization Time (s)
von Mises	55	NA
Hill48	82	3
Barlat Yld91	171	392
Barlat Yld2004-18P	155	7200

## Data Availability

The data that support the findings of this study are available on request from the corresponding author.
